# White cell count and platelet count associate with histological alcoholic hepatitis in jaundiced harmful drinkers

**DOI:** 10.1186/1471-230X-13-55

**Published:** 2013-03-26

**Authors:** Timothy Hardy, Christopher Wells, Stuart Kendrick, Mark Hudson, Christopher P Day, Alastair D Burt, Steven Masson, Stephen F Stewart

**Affiliations:** 1 The Liver Unit, Freeman Hospital, Newcastle upon Tyne NE7 7DN, UK; 2 Institute of Cellular Medicine, Newcastle University, Newcastle upon Tyne NE2 4HH, UK; 3 The Centre for Liver Disease, Mater Misericordiae University Hospital, 55 Eccles Street, Dublin 7, Irish Republic

**Keywords:** Alcoholic hepatitis, Diagnosis, Accuracy, Biopsy, Biochemical markers

## Abstract

**Background:**

Patients with suspected alcoholic hepatitis and a Discriminant Function ≥32 underwent liver biopsy to confirm the diagnosis. Of these (n = 58), 43 had histological features of alcoholic hepatitis and 15 (25%) did not.

We aimed to determine the laboratory features that differentiated those patients with a histological diagnosis of alcoholic hepatitis from those without, and assess potential clinical utility.

**Methods:**

Laboratory investigations at presentation for each of the histologically confirmed cases of alcoholic hepatitis (n = 43) were compared to those without (n = 15) to determine whether there were differences between the two groups. Univariate analysis was by Mann Whitney *U* Test and Multivariate analysis was by a stepwise approach.

**Results:**

White cell count (16.2 ± 10.5 v 6.9 ± 3.5 (× 10^9^/L); p = 0.0001) and platelet count (178 ± 81 v 98.4 ± 43 (× 10^9^/L); p = 0.0005) were higher in the patients with histological features of alcoholic hepatitis than in those without. The area under the ROC curve for AH diagnosis was estimated to be 0.83 (0.73, 0.94) and 0.81 (0.69, 0.93) for white cell count and platelet count respectively.

**Conclusions:**

Clinicians cannot accurately differentiate patients with or without alcoholic hepatitis without liver biopsy. This is critically important when deciding on specific therapies such as corticosteroids or when interpreting data from future trials in which biopsy is not mandated. In situations where liver biopsy is unsuitable or unavailable the white cell and platelet counts can be used to determine the likelihood of histological alcoholic hepatitis and guide treatment.

## Background

Alcoholic hepatitis (AH) is defined by a constellation of histological abnormalities. These consist of liver cell damage (in the form of ballooning degeneration), a neutrophil inflammatory cell infiltrate, Mallory bodies and pericellular fibrosis [1]. This fibrosis characteristically commences in the perivenular zone [2] and has a “chicken wire appearance”. These histological features are associated with a clinical syndrome of alcoholic hepatitis, but the severity of the clinical syndrome does not closely reflect the severity of the histology. The clinical syndrome can be mild with non-specific symptoms and mild liver transaminase abnormalities, or severe with jaundice, ascites and encephalopathy [3,4].

Prognosis in alcoholic hepatitis correlates with the severity of the underlying histological lesion [5,6]. In severe cases, however, this prognostic information is hard to obtain as transabdominal liver biopsy is precluded on the grounds of coagulopathy associated with the liver failure. Transjugular liver biopsy is an alternative but is not always available. In addition, it has a small but definite mortality in this population [7-9]. For this reason, prognostic scores based on clinical parameters such as the prothrombin time, bilirubin, white cell count and renal function have been developed [10,11]. These are now used in many units to make treatment decisions, particularly to decide whether to give the patient corticosteroids or not. The clinical utility of these scores means that many physicians do not confirm the diagnosis of alcoholic hepatitis with biopsy but treat according to Maddrey’s Discriminant Function (DF) [10] or the Glasgow Alcoholic Hepatitis Score [11] This has a pragmatic attraction, particularly in centres where transjugular liver biopsy is not available. There is a concern however that some cases that are treated are not alcoholic hepatitis at all but decompensated cirrhosis. The outcome of these patients when treated with steroids is not known.

In this study, our aims were to report on the frequency with which the clinical diagnosis of alcoholic hepatitis is inaccurate and to determine if there were any clinical differences between the group with or without alcoholic hepatitis on biopsy. We also sought to assess the clinical utility of any observed differences between the two groups.

## Methods

Patients with suspected alcoholic hepatitis and a DF ≥ 32 underwent liver biopsy to “confirm” alcoholic hepatitis. Most of these patients underwent biopsy prior to entry into a randomized controlled trial of antioxidants versus placebo in alcoholic hepatitis, which was carried out in the Freeman Hospital Liver Unit in Newcastle upon Tyne, UK [12]. The inclusion criteria were a recent history of alcohol excess, jaundice and a discriminant function ≥32 [13]. Exclusion criteria were active infection, spontaneous bacterial peritonitis, upper GI bleed and previous cardiac or respiratory disease [12]. All patients that fulfilled the criteria were biopsied to confirm or refute the clinical suspicion of alcoholic hepatitis [12]. Some patients were subsequently excluded because they had no histological features of alcoholic hepatitis. To increase patient numbers we have included patients subsequently admitted to our unit who were biopsied to investigate presumed alcoholic hepatitis and were found not to have the relevant histological features, using the same criteria.

Laboratory data (full blood count, serum bilirubin, liver enzymes, prothrombin time (PT), creatinine and albumin) from the time of admission were collected. From these data, the Child-Pugh Score [13] and Discriminant Function [10] were derived. Liver biopsies were reviewed, classified and staged by an expert pathologist (ADB) according to criteria published previously [1].

Descriptive statistics are provided as the mean (±SD), median and range or percentage for quantitative and qualitative variables, respectively. Comparisons between groups were performed with the Mann–Whitney *U* test. For multivariate analysis a stepwise approach was used. Fisher’s exact test was used to test the association of histological stage with presence of histological AH. A p value of < 0.05 was considered statistically significant. Receiver operating characteristic (ROC) curve analysis was used to assess the utility of White Cell Count and Platelet Count in the diagnosis of AH. IBM SPSS 17 was used to perform all analyses.

Patients gave written informed consent for entry into the trial. Ethics for the original antioxidant trial was granted by Newcastle and North Tyneside Local Ethics Committee [12]; research was conducted in compliance with the Helsinki Declaration.

## Results

The main characteristics of the 58 patients are given in Table 1. Of these, 49 patients were included from the original antioxidant study, with 9 additional patients identified subsequently. From these 58, 43 (74%) had histological features of alcoholic hepatitis and 15 were found to be cirrhotic with no histological features of alcoholic hepatitis. Of the 43 patients with histological features of alcoholic hepatitis, 35 (81%) were found to be cirrhotic. Histological features of the 58 patients are given in Table 2. Lab indices according to fibrosis stage are given in Table 3. Diagnoses of 12 patients without histological features of alcoholic hepatitis were spontaneous bacterial peritonitis, pneumonia, sepsis of unknown source or GI bleed. In the remaining three patients no clear diagnosis could be made other than end stage alcoholic cirrhosis.

**Table 1:** Characteristics of the patients of the 43 patients with AH versus 15 patients without AH

	**AH (n = 43)**	**No AH (n = 15)**	**p-value**
Age (yrs)	44.8 ± 8.5 (27–74)	47.3 ± 4.7 (41–54)	0.2997
White cell count (× 10^9^/L)	16.2 ± 10.5 (2.8–58.8)	6.9 ± 3.5 (3.1–15.1)	0.0001
Platelet Count (× 10^9^/L)	178 ± 81 (23–130)	98.4 ± 43 (34–160)	0.0005
Bilirubin (μmol/L)	431 ± 195 (143–876)	307 ± 194 (83–599)	0.1797
Alkaline Phosphatase (U/L)	179.2 ± 64 (78–317)	128.2 ± 51.5 (79–175)	0.1628
AlanineTransaminase (U/L)	41.6 ± 23.4 (11–139)	36.3 ± 19.2 (16–59)	0.1583
Prothrombin Time (secs)	26.0 ± 5.6 (20–47)	25.0 ± 6.2 (18–35)	0.6917
Child Pugh Score	12.0 ± 1.3 (9–14)	11.5 ± 2.4 (7–14)	0.8189
Discriminant Function	74.7 ± 29.6 (37–191)	76.2 ± 33.6 (34–128)	0.7954
Albumin (g/L)	28 ± 4.4 (19–40)	27.6 ± 6.2 (20–37)	0.9150
Creatinine (μmol/L)	185 ± 207 (52–1064)	130 ± 119 (52–371)	0.1653
Male/Female Ratio	1.13	2	

Plus-minus values are means + -SD. Ranges are shown in the parentheses.

**Table 2:** Histological features of the patients of the 43 patients with AH versus 15 patients without AH

	**AH (n = 43)**		**No AH (n = 15)**	
Histology				
Steatosis				
Macro	37	(86)	11	(73)
Mixed	6	(14)	4	(27)
Ballooning	43	(100)	0	(0)
Inflammation	43	(100)	0	(0)
Mallory-Denk Bodies	42	(98)	5	(33)
Fibrosis Stage				
F3	8	(19)	0	(0)
F4	35	(81)	15	(100)

Values in brackets are presented as percentages.

**Table 3:** Lab indices of the 8 patients with F3 fibrosis versus 50 patients with F4 fibrosis

	**F3 (n = 8)**	**F4 (n = 50)**	**p-value**
Age (yrs)	45.3 ± 6.3 (34–53)	45.1 ± 8.5 (27–74)	0.9552
White cell count (× 10^9^/L)	24.5 ± 17 (6.9–58.8)	13.6 ± 8.2 (2.8–44.7)	0.0084
Platelet Count (× 10^9^/L)	186 ± 101 (88–354)	165 ± 80 (34–379)	0.5519
Bilirubin (μmol/L)	403 ± 198 (143–663)	419 ± 200 (83–876)	0.8519
Alkaline Phosphatase (U/L)	179.7 ± 70 (105–317)	170 ± 64 (69–297)	0.8186
Alanine Transaminase (U/L)	43.4 ± 17.5 (23–71)	40.6 ± 23.4 (13–139)	0.5396
Prothrombin Time (secs)	29 ± 9.1 (22–47)	25.4 ± 4.8 (18–41)	0.1202
Child Pugh Score	12.5 ± 1 (11–14)	11.9 ± 1.5 (7–14)	0.2477
Discriminant Function	89.9 ± 48.6 (51–191)	72.4 ± 25.4 (34–151)	0.1523
Albumin (g/L)	28 ± 4.8 (24–38)	28 ± 4.7 (19–40)	0.9507
Creatinine (μmol/L)	209 ± 157 (52–546)	173 ± 206 (52–1064)	0.6627

Plus-minus values are means + -SD. Ranges are shown in the parentheses.

White cell count (16.2 ± 10.5 v 6.9 ± 3.5 (×10^9^/L); p = 0.0001) and platelet count (178 ± 81 v 98.4 ± 43 (× 10^9^/L); p = 0.0005) were higher in the patients with histological features of alcoholic hepatitis than in those with none. Both clinical parameters remained significant on multivariate analysis.

**Figure 1:**
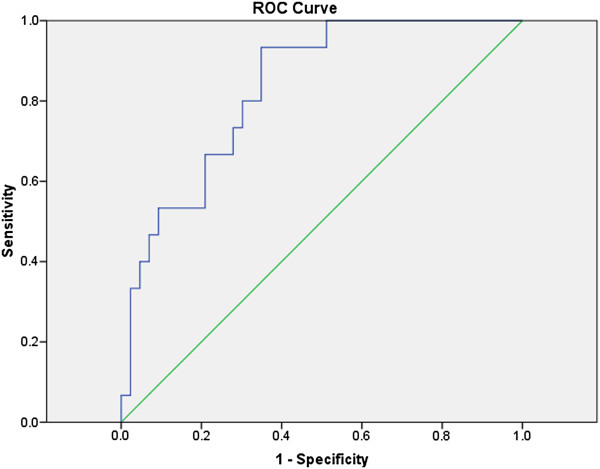
**White cell count for diagnosis of AH.** The area under the ROC curve is shown for the performance of the White Cell Count for discriminating AH from no AH diagnosis.

**Figure 2:**
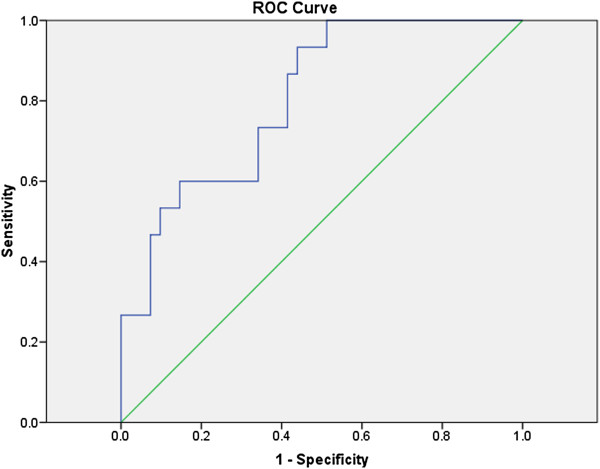
**Platelet count for diagnosis of AH.** The area under the ROC curve is shown for the performance of the Platelet Count for discriminating AH from no AH diagnosis.

Using the area under the receiver operating characteristic (ROC) curve approach, we next calculated potential cut-off values to separate patients with AH from those without AH based on White Cell Count and Platelet Count. The area under the ROC curve (AUC) was estimated to be 0.83 (0.73, 0.94) (Figure 1) for white cell count and 0.81 (0.69, 0.93) for platelet count (Figure 2).

We chose a cut off of 10.75 × 10^9^/L for white cell count and 147.5 × 10^9^/L for platelet count for further analysis. A white cell count of > 10.75 × 10^9^/L had a sensitivity of 65% and a specificity of 93% for detecting AH (PPV 97% and NPV 48%). A platelet count of >147.5 × 10^9^/L had a sensitivity of 56% and a specificity of 93% (PPV 96% and NPV 56%). A white cell count > 10.75 × 10^9^/L and a platelet count of >147.5 × 10^9^/L was seen in 19 patients and had a sensitivity of 44% and a specificity of 100% for detecting AH (PPV 100% and NPV 38%).

We then chose a cut off of 5.95 × 10^9^/L for white cell count and 86 × 10^9^/L for platelet count to determine a cut off below which we could accurately detect patients without AH. A white cell count of <5.95 × 10^9^/L had a sensitivity of 47% and a specificity of 93% for detecting patients that did not have AH (PPV 70% and NPV 83%). A platelet count of <86 × 10^9^/L had a sensitivity of 47% and a specificity of 93% (PPV 64% and NPV 83%). A white cell count <5.95 × 10^9^/L and a platelet count <86 × 10^9^/L had a sensitivity of 45% and a specificity of 98% for detecting those patient without AH (PPV 87.5% and NPV 83%).

**Figure 3:**
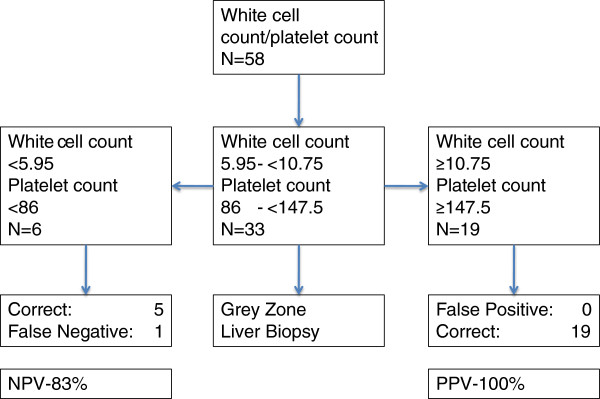
Prediction of alcoholic hepatitis by white cell count and platelet count.

By using both upper and lower cut-off values we were able to develop an algorithm (Figure 3), which could be used to avoid (transjugular) liver biopsy in 24/58 patients while maintaining good diagnostic accuracy.

Fishers exact test was non significant (p = 0.08) in determining an association between fibrosis stage and presence of AH on biopsy. Only WCC remained statistically significant (p = 0.0084) when groups were analysed according to fibrosis stage (see Table 3).

## Discussion

For pragmatic reasons, treatment decisions are often made without liver biopsy in those patients with suspected severe alcoholic hepatitis. These reasons include the availability and safety of transjugular liver biopsy. They also reflect the trials of therapy in severe alcoholic hepatitis. While there have been positive trials of steroids where all patients were biopsied [14], there have also been positive studies where biopsy was not a requirement [15]. This leaves the hepatology community in two camps regarding the importance of biopsy, and this is reflected in practice guidelines where precise indications for liver biopsy are not well established [16-18]. Recent EASL Guidelines recognise that many centres rely on clinical criteria, and do not consider biopsy as routine practise. However the guidance includes biopsy in the therapeutic algorithm and recommends that it should be considered in high risk patients according to prognostic assessment with Maddrey’s for example [18].

We wanted to know the frequency with which a patient with a clinical presentation suspicious of alcoholic hepatitis and a DF > 32 had alcoholic hepatitis on liver biopsy. In addition, we wanted to know whether laboratory factors could be used to improve accuracy.

We found that 74% of patients with a high clinical suspicion of alcoholic hepatitis had this confirmed histologically. Had the group not undergone biopsy, 25% (n = 15) of the total number would have undergone specific treatment without having the clinical condition the treatment was specifically targeted to. This supports the conclusions of the EASL guideline for the use of liver biopsy in the setting of alcoholic hepatitis.

When we analysed the laboratory features at presentation we found two interesting findings. The first is that the group with alcoholic hepatitis on liver biopsy had a higher platelet count (178 ± 81 v 98.4 ± 43 (× 10^9^/L); p = 0.0005) than those without alcoholic hepatitis. This is likely to be due to the fact that patients with no AH have more advanced fibrotic liver disease with portal hypertension leading to decompensation. The second was that the group with alcoholic hepatitis on liver biopsy had a higher mean white cell count (16.2 ± 10.5 v 6.9 ± 3.5 (× 10^9^/L); p = 0.0001). Using a cut off value of >10.75 × 10^9^/L for white cell count and >147.5 × 10^9^/L for platelet count, accurately diagnoses AH with a combined specificity 100%. A lower boundary of <5.95 × 10^9^/L and <86 × 10^9^/L for white cell count and platelet count respectively, accurately rules out AH as a diagnosis with a combined sensitivity of 98%. There are two important clinical ramifications of this finding. The first is that there should be suspicion regarding labelling patients with a normal white cell count as alcoholic hepatitis. The second is that alcoholic hepatitis is an inflammatory condition and a high white cell count is entirely in keeping with this. A raised white cell count should not therefore be used as a reason not to give treatment because of concerns about infection when there are no other features of overt sepsis.

Together these laboratory tests may help the clinician to make a decision about whom to biopsy; particularly if this means sending them to another hospital. We would advise caution in making a diagnosis of acute alcoholic hepatitis, regardless of the clinical presentation in any patient with a normal white cell count, particularly if this is associated with a very low platelet count.

The majority of the patients in our study were cirrhotic 50/58 (86%). As expected, all 15 of the patients that were found to not have alcoholic hepatitis on liver biopsy were cirrhotic. These are the patients that progressed to decompensation due to very advanced liver disease rather than superimposed hepatitis. 8/43 patients with AH were non-cirrhotic; all of these were F3. When we compared laboratory indices between the cirrhotic patients and the non-cirrhotic patients we found the only statistically significant finding to be the difference in average WCC. This is largely due to the fact that the F4 cohort contained all the patients that did not have histological alcoholic hepatitis.

Our results suggest that in future clinical trials where biopsy is not mandated, 25% of patients maybe falsely included. This clearly has implications for the validity and reliability of data testing specific therapies in patients whom may not suffer from the disease they are targeted to; this is reflected in the EASL Clinical Practical Guideline in Alcohol whereby performing liver biopsy prior to trial commencement is recommended.

What is needed now are data from large prospective studies where biopsy is included in the diagnostic algorithm but where patients are included in the therapeutic study regardless of the result. This will help to elucidate the response of the patient presenting with the alcoholic hepatitis syndrome but a non-confirmatory biopsy to standard of care treatment. Should these studies show that treatment is detrimental to this group, biopsy should in future be mandated prior to commencing treatment. In situations where transjugular biopsy is impractical or simply unavailable the surrogate markers of white cell count and platelet count may prove to be of great use in determining which patients receive treatment.

## Conclusions

In conclusion, we have shown that the diagnosis of alcoholic hepatitis is difficult on clinical grounds; we have also found two clinical parameters that can assist the clinician with the diagnosis. This may in time prove to be very useful when determining treatment strategies.

## Competing interests

The authors declare that they have no competing interests.

## Authors’ contributions

TH: acquisition of data, analysis and interpretation of data, drafting the manuscript. CW, SK, MH, CP: acquisition of data. AB: acquisition of data, analysis and interpretation of data (liver biopsies). SS: Conception and design, analysis and interpretation of data, drafting and revising for important intellectual content. All authors read and approved the final manuscript.

## Pre-publication history

The pre-publication history for this paper can be accessed here:

http://www.biomedcentral.com/1471-230X/13/55/prepub
